# Quality Control of 11 Cannabinoids by Ultraperformance Liquid Chromatography Coupled with Mass Spectrometry (UPLC-MS/MS)

**DOI:** 10.1155/2023/3753083

**Published:** 2023-08-10

**Authors:** Ashraf Duzan, Desiree Reinken, Mufeed M. Basti

**Affiliations:** ^1^Wingate University School of Pharmacy, Wingate University, Wingate, NC, USA; ^2^Applied Science and Technology Department, North Carolina State University of Agriculture and Technology, Greensboro, NC, USA; ^3^Department of Pharmaceutical Sciences, College of Pharmacy, Health Professions Division, Nova Southeastern University, Fort Lauderdale, FL, USA; ^4^University of Colorado College of Nursing, University of Colorado Anschutz Medical Campus, University of Colorado, Boulder, CO, USA

## Abstract

**Objective:**

Cannabinoid extraction from *Cannabis sativa* L. (hemp) for nonmedical purposes has become popular in the United States. Concerns, however, have been raised regarding the accuracy of the labels for cannabinoid levels in the commercial products.

**Methods:**

In this study, we developed rapid, sensitive, selective, accurate, and validated liquid chromatography-tandem mass spectrometry for the quantification of cannabinoids. The methods are for determining 11 cannabinoids in cannabis (hemp) extracted in oil form, and we investigated the accuracy of the labeling and thermal stability regarding the cannabinoids on 17 oil cannabis samples.

**Results:**

In the UPLC chromatogram, we see a good resolution and there is no matrix effect and the accuracy were 98.2% to 102.6%, and the precision was 0.52%–8.18%. The linearity of the calibration curves in methanol was with a regression *r*^2^ ≥ 0.99. The lowest of detection (LOD) was 5–25 ng/mL, and the limit of quantification (LOQ) was 10–50 ng/mL. The study showed that only 30% of the commercial samples were within the acceptable range of +/−10% compared to the labeled ingredient concentrations. The thermal stability test profile showed a change in the concentration of cannabinoids in each sample at 37°C for one week, with an average loss of cannabinoids up to 15%.

**Conclusion:**

The validated method proved to be selective, accurate, and precise, with acceptable linearity within the calibration range with no matrix effect. The stability profile data indicated that high temperatures could change the quality of commercial samples.

## 1. Introduction

**Figure 1:**
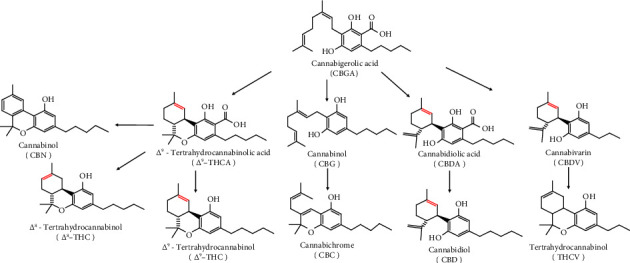
Schematic representation of chemistry features of the most common cannabinoids in *Cannabis sativa* L. plant. They are CBGA: cannabigerolic acid, Δ^9^-THCA: delta-9-tetrahydrocannabinolic acid, CBDA: cannabidiolic acid, CBD: cannabidiol, Δ^8^-THC: delta-8-tetrahyrocannabinol, CBN: cannabinol, CBDV: cannabidivarin, Δ^9^-THC: delta-9-tetrahydrocannabinol, CBC: cannabichrome, CBG: cannabigerol, and THCV: tetrahydrocannabivarin [6, 7].

*Cannabis sativa* (marijuana) products are widely consumed products for recreational purposes nationwide, and using as medicinal forms is currently under scrutiny [1–3]. Delta-9-tetrahydrocannabinol (Δ^9^-THC) is the primary psychoactive compound in cannabis preparations and is converted to other analogs by several enzymes in the liver and gut microbiota [4, 5]. Cannabinoids are a class of chemical compounds synthesized in plants by a very complex enzymatic system that converts one analog to another. For example, cannabigerolic acid (CBGA) is converted to cannabigerol (CBG) through decarboxylation (Figure 1) [6, 7]. Despite research breakthroughs over the last three decades, cannabis plants remain classified by the Food and Drug Administration (FDA) as a Schedule I Controlled Substance under the Controlled Substances Act of 1970. In states that have not passed medicinal marijuana or recreational marijuana legislation but do allow hemp products for commercial sale, Δ^9^-THC must be at or below the concentration of 0.3% [8–10]. Cannabidiol (CBD) and Δ^9^-THC are isolatable phytocannabinoid molecules used to treat cancer [11, 12].

*Cannabis sativa L* (cannabis, hemp, or marijuana) is subspecies hybrid, and the extracts are generally classified into two main categories: full- and broad-spectrum products. Broad-spectrum products contain primary cannabinoids with various concentrations extracted from cannabis and have bioactivity or therapeutic effects. The full-spectrum extract contains primary cannabinoids such as Δ^9^-THC at the level below the detection level for most laboratory testing purposes. The main active ingredient in these products is CBD. CBD is white to slightly off-white in color, crystalline, and has a mild (almost unnoticeable) smell/odor. CBD can be obtained by extraction and distillation from plants such as *Cannabis sativa* via inflorescence or synthesized by stereoselective laboratory techniques. CBD-containing products have emerged in high demand in many states since they are marketed in herbal, cosmetic, and pseudopharmaceutical forms. These products use current good manufacturing practices (GMPs/CGMPs) and have been considered to validate the concentrations of CBD and Δ^9^-tetrahydrocannabinol (Δ^9^-THC) in different products ranging among topical oils, tinctures, gummies, soft-gel lozenges, and smokables [13–15].

Cannabinoids have been categorized into many subclasses. Each subclass contains cannabinoids and derivatives according to their chemical features. These subclasses include CBGA, Δ^9^-tetrahydrocannabinolic acid (Δ^9^-THCA), cannabidiolic acid (CBDA), CBD, Δ^8^-tetrahydrocannabinol (Δ^8^-THC), cannabinol (CBN), cannabidivarin (CBDV), Δ^9^-THC, cannabichromene (CBC), CBG, and tetrahydrocannabivarin (THCV) [16].

This study aims to develop a robust ultraperformance liquid chromatography-tandem mass spectrometry (UPLC-MS/MS) quantitative laboratory analysis of cannabinoids. Such analysis is paramount for quality control processes that can be used to quantify and verify cannabinoid levels in commercially available products. Moreover, the effects of temperature were investigated to quantify the changes in cannabinoid levels.

## 2. Materials and Methods

### 2.1. Chemicals, Samples, and Reagents

The certified standards for CBGA, CBG, CBDA, CBD, Δ^8^-THC, CBDV, CBN, Δ^9^-THC, and Δ^9^-THCA were purchased from Sigma-Aldrich (St. Louis, MO, USA), and CBC was procured from Cerilliant Corporation (Round Rock, Texas). Mass spectrometry-grade formic acid, methanol, and acetonitrile (methyl cyanide) were procured from Fisher Scientific (Waltham, MA). Acquity ultra-high-performance liquid chromatography (UPLC) BEH C18 analytical column and VanGuard precolumn for chromatography were obtained from Waters Corp., Waltham, MA. Cannabinoid samples were of oil form containing full-spectrum containing either CBD or Δ^8^-THC in commercial samples and were obtained from local hemp stores at Wingate, NC, USA. These products were stored at −20°C until further analysis.

### 2.2. Instrumentation and Data Processing

High-performance liquid chromatography-tandem mass spectrometry was used. The Acquity Classic UPLC® system consisted of a Waters sample manager, binary solvent manager, cooled sample trays, integrated column heater, and degasser. The UPLC system was equipped with binary system pumps, an autosampler, a built-in degasser, and a column heater coupled with a Xevo TQ MS detector. A sample loop in the injection mode was used to inject 10 *µ*L samples. MassLynx software (version 4.2) was used to collect the data processed using TargetLynx (version 4.2). Cannabinoids in eluted samples were quantified by using a UPLC-MS/MS system consisting of a quadrupole time-of-flight mass spectrometer system (Q-Tof-MS/MS) (Waters Xevo TQ-XS with Z-spray ionization and step wave source optimization). The equipment calibration and detector validation processes were performed daily using octreotide and standard solutions with the integrated Intelli Start procedure of the MassLynx V4.2 system software. The resulting mass spectrometric parameters were determined using argon collision gas for collision-induced dissociation (CID), coupled with the exact mass measurement with time-of-flight (ToF) with tandem mass spectrometry (MS/MS) transitions where the analytes and standards were monitored in the positive or negative ion modes. The exact mass was used to determine the elemental composition of the target molecules.

### 2.3. Liquid Chromatographic (LC) Conditions

Analytes were separated on an Aquity UPLC BEH C18 analytical column (2.1 × 100 mm, 1.7 *µ*m particle size, and 130 Ǻ pore size) preceded by an Acquity UPLC BEH C18 VanGuard precolumn (2.1 × 5 mm, 130 Ǻ). The flow rate was kept at 0.5 mL/min, and 5 *µ*L of the sample was injected into the column. The autosampler was maintained at 10°C throughout the analysis, and the analytical column was maintained at 45°C. The mobile phases consisted of 0.1% formic acid in water (A) and 0.1% formic acid in acetonitrile (B). A linear gradient was used to separate the analytes over a run time of 13 min. The gradient conditions were as follows: 50% A for 1 min; 100% B for 8 min; 50% B for 3 min; and equilibration of the column for 1 min.

### 2.4. Mass Spectrometry Conditions

Quadrupole time-of-flight tandem mass spectrometer system (Waters SYNAPT G2-Si Q-ToF) parameters were optimized using tandem MS (MS/MS) ions for each standard solution of cannabinoids in the positive and negative modes. The most common cannabinoids have similar precursor (parent) ions but different products (daughters). The method was validated and followed the FDA guidelines. Electrospray ionization (ESI) in the positive and negative modes was used to quantify the analytes' tandem MS/MS transitions. Major analyte-specific mass spectrometer settings used during the analysis in the positive mode for protonated precursors (M + H)^+^ were selected for CBD, CBG, CBDV, THCV, CBN, Δ^8^-THC, Δ^9^-THC, and CBC. The deprotonated precursors (M-H)^−^ were chosen for CBDA, Δ^9^-THCA, and CBGA. A total ion chromatogram (TIC) was used to quantify the analytes (Table 1). Mass spectrometry parameters included capillary voltage of 1.50 kV, collision gas flow of 0.15 mL·min^−1^, extractor voltage of 3 V, desolation temperature of 500°C, source temperature of 150°C, and desolation gas flow of 1000 L/h, and the scan MS was 50–1200 m/z. The quantification was operated in the MSMS mode. For MS^E^ experiments, one acquisition function with different collision energy ramps was used for additional MS/MS experiments with electrospray ionization (ESI). The system was organized with the Analyst 1.6.3 software, and data were collected by MultiQuant 3.0.2 system. Data were processed using TargetLynx software (within MassLynx).

### 2.5. Validation of the Bioanalytical Method

This validation method followed the general guidelines for bioanalytical method development issued by the US FDA [17]. The limits of detection and quantification, linearity, precision, accuracy, recovery, and matrix effect tests were evaluated and validated. Oil of English ivy plant (0% CBD) was used as the matrix to measure the recovery percentages.

#### 2.5.1. Standard and Quality Control Samples

A standard stock solution of the 11 cannabinoid solutions was prepared in methanol at a 1 mg/ml concentration. Calibration curves and quality control samples were included for each run. The area under curve (AUC) ratios were recorded and plotted against the concentrations of the standards. Five replicates were used for each of the six stock solutions of each of the 11 cannabinoids with the final concentrations of 0, 50, 100, 150, and 200 ng/ml. All spiked samples and stock solutions were stored at −20°C. The lowest of detection (LOD) was used where the signal-to-noise ratio, S/N, was higher than 3, whereas the limit of quantification (LOQ) was established at a signal-to-noise ratio S/N ≥ 10. The coefficient of variation (CV%) was ≤20%. The acceptance criteria for quality control samples (QCs) include the limit of quantification (LOQ), the middle of quantification or detection (MOQ/MOD), and the highest of quantification or detection (HOQ/HOD) at RSD ≤ 15%.

#### 2.5.2. Extraction Procedure

Samples were extracted from cannabis using the solid-liquid method. The weights of the cannabis samples, such as flower, crude extract, tincture, or cream on clean and dry paper, were 0.1–0.5 g. The flower sample was ground into a fine powder using a mortar and pestle. Five milliliters of acetonitrile (LCMS grade) were added to the sample in the centrifuge tube. Gen Power 125 was used to mix the powder with the solvent for 20 min and then the mixture was vortexed for 3 min. The mixture was sonicated for 15 min and centrifuged at 13000 rpm for 15 min. The supernatant was then transferred into a separate tube. The extraction with ACN was repeated 4 times, and all the fractions (20 ml) were mixed. The extract went through a dilution factor of 100 and was vortexed for 1 min and was then filtered using a 0.45 *µ*m PTFE filter unit. Samples were stored at 4°C for analysis. Before transferring 100 *µ*L of the extract to the LCMS vial, the extract was centrifuged for 5 min.

#### 2.5.3. Matrix Effect, Recovery, Accuracy, and Stability Tests

For analysing matrix effects, the oil of English ivy plant was used as the matrix with 0% cannabinoids. The AUCs of 11 standard cannabinoids were spiked and quantified and compared to the spiked solvent (ACN) at the three quality control concentrations. The AUC was also used to measure the recovery percentages after extraction. In five replicates, accuracy was evaluated relative to the calibration curve at three different concentrations (LOQ, MOC, and HOQ). The effect of temperature on the stability of the samples was investigated for all the analytes. After seven consecutive days, the concentration changes were recorded at different temperatures (−20, 4, 25, 37°C). The experiments were replicated (*n* = 5).

## 3. Results

### 3.1. LC-MS/MS Method Development

**Figure 2:**
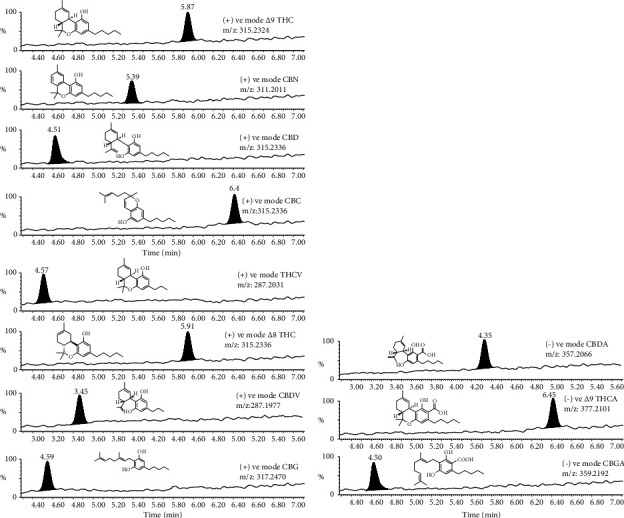
The chromatograms of water that was spiked with cannabinoids at concentration corresponding to the limit of quantification (LOQ). The cannabinoids were observed in the positive MS mode (a) and in the negative MS mode (b). The chemical structures and molecular weights of the cannabinoids that were determined via UPLC-MS/MS are also shown.

Figure 1 shows the schematic presentation of the eleven cannabinoids analyzed in this report, and Table 1 lists their retention times (RTs).  Figure 2 shows the chromatograms of water spiked with cannabinoids at a LOQ concentration of 5 ng/ml where the MS mode of detection was positive (2(a)) or negative (2(b)); m/z(s) were determined by UPLC-MS/MS, and the chemical structure is also shown in Table 1. The degree of the linearity for the calibration curve was within the acceptable range (*r*^2^ = 0.99) [18] (Table 1).

### 3.2. Precision and Accuracy

The precision and accuracy of the method were measured by analyzing data of the LOQ, MOQ/MOD, and the HOQ/HOD of the 11 cannabinoids. These were prepared and evaluated using five replicate points within the calibration curve between 50 and 200 ng/mL. The correlation coefficient (*r*^2^) was determined to be ≥ 0.99 (Figure S1).

### 3.3. Extraction Recovery and the Matrix Effect

**Figure 3:**
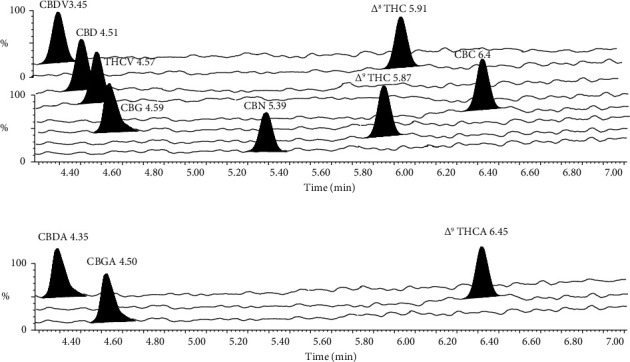
Extracted ion chromatograms in the positive (a) and negative (b) ionization modes of the matrix solution. UPLC-MS/MS chromatogram of analytical standards mixture at 10 ng/ml of the limit of quantification (LOQ).

The method assessed the extraction recovery and the matrix effect of 11 cannabinoid analytes at LOQ, MOQ/MOD, and HOQ/HOD for all analytes (*n* = 5). The extraction recovery ranged from 86.0 to 110.88%. The matrix effect was detected for the 11 cannabinoids with 3 replicates in the range of 91.98–111.44% (Table S1, Figure 3).

### 3.4. Application of the Assay to Quantify Cannabinoids and the Stability Profile of 17 Commercial Samples

Table 2 lists the comparison between the experimental and labeled cannabinoid concentration for 17 commercially available products. The assay quantified the cannabinoids in each of these commercial products. Tables 2 and 3 present the difference in cannabinoid concentrations (mg/mL) after 7 days at four different temperature conditions (−20, 4, 25, and 37°C). The data presented are the mean values with the standard error of the mean (SEM), and superscripts indicate the significance of the comparisons among the groups.

Regarding the calculation of the concentrations, it is important to clarify that the concentrations were determined using the calibration curves generated in the assay. The calibration curves were created based on the best-fit linear regression method, as shown in Figure S1. To calculate the cannabinoid concentrations in the samples, the software (TargetLynx) integrated within MassLynx was employed. The software utilizes the calibration curves to determine the concentration of cannabinoids in the samples based on their respective peak areas. This method allows for accurate quantification of cannabinoids by incorporating the calibration curves developed using the UPLC-MS/MS method. The label contained the concentration of the major cannabinoids in these products, ranging from 1.3 to 95 mg/mL, indicating a high percentage of error (% difference) (Table 2) in some cases. The US Pharmacopeia (USP) guidelines suggest that the experimental results should be within +/−10% of the reported data in the product. These products' thermal stability in terms of the level of cannabinoids was assessed as they were stored at different temperature conditions as follows: −20, 4, 25, and 37°C for one week (Table 3).

## 4. Discussion

Figure 1 shows that the method was very sensitive compared to published reports [19, 20]. Table 1 also lists the LOD for the 11 cannabinoids. The peak intensities and UAC of the analytes were the same whether the acquired chromatogram was obtained in the blank or matrix conditions.

Table 2 presents the comparison between the labeled cannabinoid concentrations and the experimental concentrations obtained through quantification using the UPLC-MS/MS method for 17 commercially available cannabinoid products. The labeled concentrations of major cannabinoids in these products were provided by the manufacturer, ranging from 1.3 to 95 mg/mL. The percent difference (% difference) between the labeled and experimental concentrations is also reported in the table. It is important to note that the US Pharmacopeia (USP) guidelines suggest that experimental results should fall within +/−10% of the reported data on the product labels. Based on this guideline, several products in Table 2 show a high percentage of error (% difference) between the labeled and experimental concentrations. For example, sample 2 (THC) exhibits a −60.0% difference, indicating a lower experimental concentration compared to the labeled value. On the other hand, samples 3, 4, 5, and 13 (CBD and THC) show positive percent differences, indicating higher experimental concentrations than the labeled values. Regarding sample 10, it is identified as a noncannabinoid sample in Table 2, which explains why the experimental concentration is reported as 0.0 mg/mL. The presence of a noncannabinoid sample in the dataset can provide valuable information for assessing the specificity and accuracy of the quantification method, as it should ideally yield a nondetectable result.

The accuracy and precision data were within the acceptance criteria, with a precision of ≤15% and accuracy within ±15%. The actual accuracy of different analytes, shown in Table 4, was between 98.29 and 110.27% of their points for calibrators. The precision was between 0.52 and 8.18% (Table 4).

Likewise, no matrix effect was detected, and it was within the acceptable range for the 11 cannabinoids. Table 1 indicates that only 30% of the samples were within the acceptable range. Our explanation for samples whose experimental values did not match the labels is human error in their analysis or inadequate storage and/or transportation conditions. The results (Table 3) showed that at 37°C, CBD and THC concentrations could change by more than 10% on average. Temperature is a significant factor that can change the concentrations of some isomers or acid forms of cannabinoids. For example, oxidation and reduction may convert Δ^9^-THCA to CBNA and Δ^9^-THC to Δ^8^-THC, and decarboxylation processes may convert CBGA to CBG, CBDA to CBD, and Δ^9^-THCA to Δ^9^-THC20 (Figure 1).

## 5. Conclusion

With the increase in consuming cannabis (hemp) products in the market, we developed a new analytical method to analyze cannabinoid-containing commercial products to determine whether they meet the current regulatory requirements to protect consumers' health. UPLC-MS/MS has become a successful technique for analyzing and measuring cannabinoids with high sensitivity and precision. The used UPLC-MS/MS in our study was developed and validated by the FDA. The validation met the acceptance criteria, including sensitivity, speed of analysis within 13 min, accuracy, precision, and recovery. Chromatographic separation and ion extraction by MS are powerful tools with good sensitivity and resolution for quantifying 11 cannabinoids. The lowest of the quantitation reported was very sensitive to low concentrations of the 11 tested cannabinoids (∼5 ng/mL). The labels of seventeen cannabinoid-containing commercial samples were investigated using our validated LC-MS/MS method. The results showed that only 30% of the samples met the acceptance range. Our temperature-stability tests indicate that 4°C is a good standard temperature to maintain the cannabinoid products under safe conditions. The temperature in combination with humidity, light, packaging materials, and excipient materials will be the subject of our future investigation on cannabinoid products such as oil, vapor, cream, tincture, and cigarettes to increase the standardized laboratory testing for the quantification of all cannabis products.

## Figures and Tables

**Table 1:** Statistical analysis of a six-point calibration curve from 1 ng/mL to 200 ng/mL, with five replicates for calibration standards and quality control (QC) standards for the 11 cannabinoid mixture. Tandem mass spectrometry (MS/MS) transitions calibration range results for potency. The table shows the quantitative abilities of this method for each analyte.

Names	Retention time (min)	MS/MS ion transitions	Concentration ranges (ng/mL)	Linearity (*R*^2^)	LOD	LOQ
Δ^9^-THCA	6.4	357.2101 ➔ 313.2145	0–200	0.990	10	25
CBDA	4.3	357.2066 ➔ 245.1538	0–200	0.995	5	10
CBGA	4.5	359.2192 ➔ 341.2126	0–200	0.992	5	10
CBG	4.5	317.2470 ➔ 193.1223	0–200	0.993	10	50
CBD	4.6	315.2336 ➔ 259.1668	0–200	0.996	10	25
THCV	4.5	287.2031 ➔ 165.0929	0–200	0.998	10	25
CBN	5.3	311.2011 ➔ 223.1130	0–200	0.997	5	10
Δ^8^-THC	5.91	315.2336 ➔ 193.1242	0–200	0.993	5	10
Δ^9^-THC	5.8	315.2324 ➔ 259.1705	0–200	0.992	10	25
CBC	6.4	315.2336 ➔ 193.1242	0–200	0.991	25	50
CBDV	3.4	287.19771 ➔ 65.0892	0–200	0.992	5	10

LOD, limit of detection; LOQ, limit of quantification. Values of linearity refer to the linear range.

**Table 2:** Quality control of 17 commercial samples of cannabinoids. Results of the quantification of cannabinoids by UPLC-MS/MS.

Samples	Types of cannabinoids	Label concentration (mg/mL)	Experimental concentration (mg/mL)	Percent difference (%)
1	CBG	15.5	15.1	−2.3
2	THC	3.5	1.4	−60.0
3	CBD	41	73.3	+78.7
4	CBD	58.9	110.3	+87.2
5	THC	3.2	6.2	+93.7
6	CBD	0.0	0.0	0.0
7	CBD	95	109.8	+15.5
8	CBD	71	98.6	+38.8
9	THC	1.7	2.1	+28.8
10	THC	0.0	0.0	0.0
11	CBD	3.6	5.8	+61.1
12	CBG	25	33.2	+32.8
13	CBD	33.3	60.3	+81.0
14	CBD	64	66.6	+4.0
15	CBD	6.0	6.04	+0.66
16	CBD	1.6	1.9	+18.7
17	THC	1.3	1.7	+30.7

**Table 3:** Difference in cannabinoid concentration (mg/mL) after 7 days at four different temperature conditions.

Samples	C (mg/mL)	−20°C	4°C (mg/mL)	RT (mg/mL)	37°C (mg/mL)
1	15.10	15.02 ± 0.0	14.4 ± 0.23	14.6 ± 0.31	13.5 ± 0.20
2	1.40	1.02 ± 0.01	1.0 ± 0.41	1.02 ± 0.22	0.92 ± 0.10
3	73.30	73.02 ± 0.10	73.0 ± 0.12	73.02 ± 1.00	72.02 ± 0.10
4	110.30	110.02 ± 0.10	110.0 ± 0.12	110.00 ± 0.14	109.22 ± 0.14
5	6.20	6.12 ± 0.10	6.00 ± 0.12	6.02 ± 0.11	5.62 ± 0.31
6	0.00	0.00 ± 0.00	0.0 ± 0.10	0.1 ± 0.13	0.20 ± 0.31
7	109.80	109.01 ± 0.10	109 ± 0.10	109.10 ± 0.23	109.4 ± 0.20
8	98.60	98.12 ± 0.30	98 ± 1.20	98.22 ± 0.20	95.2 ± 0.21
9	2.20	2.12 ± 0.30	2.31 ± 1.10	2.00 ± 1.00	2.51 ± 1.30
10	0.00	0.00 ± 0.00	0.00 ± 0.00	0.12 ± 0.10	0.00 ± 0.00
11	5.80	5.82 ± 1.00	5.72 ± 0.20	5.75 ± 2.00	5.01 ± 2.10
12	33.20	33.2 ± 3.00	33.00 ± 0.10	32.21 ± 1.02	31.22 ± 0.01
13	60.30	60.2 ± 2.00	60.00 ± 2.20	60.00 ± 1.30	59.02 ± 0.30
14	66.60	66.00 ± 1.30	66.01 ± 0.10	66.2 ± 0.20	63.11 ± 0.10
15	6.00	6.05 ± 0.31	6.00 ± 0.12	5.82 ± 2.00	5.02 ± 2.10
16	1.90	1.92 ± 1.60	1.90 ± 2.50	1.72 ± 0.24	1.60 ± 1.30
17	1.75	1.72 ± 0.10	1.82 ± 0.12	1.52 ± 0.10	1.22 ± 0.34

Difference in concentration indicate the significance in the comparison among the groups with 3 replicates.

**Table 4:** Precision and accuracy of the determination of cannabinoids in the samples (*n* = 5).

Nos.	Compounds	LOQ = 50 ng/mL (*n* = 5)			MOQ = 100 ng/mL (*n* = 5)			HOQ = 200 ng/mL (*n* = 5)		
		Mean	RSD (%)	Acc (%)	Mean	RSD (%)	Acc (%)	Mean	RSD (%)	Acc (%)
1	Δ^9^-THCA	49.14	2.38	98.29	101.56	1.25	101.5	201.48	0.52	100.74
2	CBDA	49.9	3.71	99.81	100.27	0.7	110.27	200.81	1.22	100.4
3	CBGA	49.81	3.8	99.62	100.78	0.88	100.78	201.03	0.98	100.51
4	CBG	50.17	1.4	100.34	100.51	1.44	100.51	200.24	0.66	100.12
5	CBD	50.96	2.27	101.92	101.06	2.52	101.01	200.73	0.52	100.36
6	THCV	50.19	1.02	100.38	100.24	0.91	100.24	200.73	0.58	100.36
7	CBN	49.95	8.18	99.91	98.91	3.2	98.57	199.07	2.91	99.53
8	Δ^8^-THC	50.79	5.46	101.58	102.49	5.26	102.49	204.07	3.62	102.03
9	Δ^9^-THC	49.34	4.6	98.6	103.6	6.7	103.6	201.48	3.6	100.74
10	CBC	49.87	7.31	99.74	99.68	4.05	99.68	198.99	1.51	99.49
11	CBDV	51.3	2.86	102.6	99.99	2.17	99.93	199.24	1.03	99.62

LOQ: lower of quantification, MOQ: middle of quantification, HOQ: high of quantification, RSD: relative standard deviation, Acc: accuracy, and %: percent.

## Data Availability

The data used to support the findings of this study are included with the supplementary information files..

## Supplementary Materials

Supplementary Materials Figure S1: calibration curves for 11 different target cannabinoids. In our study, we established calibration curves for each of the 11 target cannabinoids using a best-fit linear regression approach for quantification. The calibration curves were constructed based on known concentrations of standard solutions of each cannabinoid. These calibration curves served as a crucial reference for accurately determining the concentrations of cannabinoids in the samples analyzed. Table S1: matrix effect and recovery effect of cannabinoids in samples. Table S1 presents the matrix effect and recovery effect of various cannabinoids in the samples, with each value representing the mean percentage (*n* = 5) obtained during the analysis. The matrix effect indicates the interference of the sample matrix on the analyte's response, while the recovery effect represents the efficiency of the extraction method in quantifying the cannabinoids accurately. For each cannabinoid, three levels of concentration were considered: LOQ, limit of quantification; MOQ, midpoint of quantification; and HOQ, highpoint of quantification.
