# Supplementary material for: Quality Control of 11 Cannabinoids by Ultraperformance Liquid Chromatography Coupled with Mass Spectrometry (UPLC-MS/MS)
Source: J Anal Methods Chem. 2023 Aug 10;2023:3753083. doi: 10.1155/2023/3753083 (PMC10435299; doi:10.1155/2023/3753083)
Supplement: Supplementary Materials — Figure S1: calibration curves for 11 different target cannabinoids. In our study, we established calibration curves for each of the 11 target cannabinoids using a best-fit linear regression approach for quantification. The calibration curves were constructed based on known concentrations of standard solutions of each cannabinoid. These calibration curves served as a crucial reference for accurately determining the concentrations of cannabinoids in the samples analyzed. Table S1: matrix effect and recovery effect of cannabinoids in samples. Table S1 presents the matrix effect and recovery effect of various cannabinoids in the samples, with each value representing the mean percentage (n = 5) obtained during the analysis. The matrix effect indicates the interference of the sample matrix on the analyte's response, while the recovery effect represents the efficiency of the extraction method in quantifying the cannabinoids accurately. For each cannabinoid, three levels of concentration were considered: LOQ, limit of quantification; MOQ, midpoint of quantification; and HOQ, highpoint of quantification. [file 3753083.f1.docx]

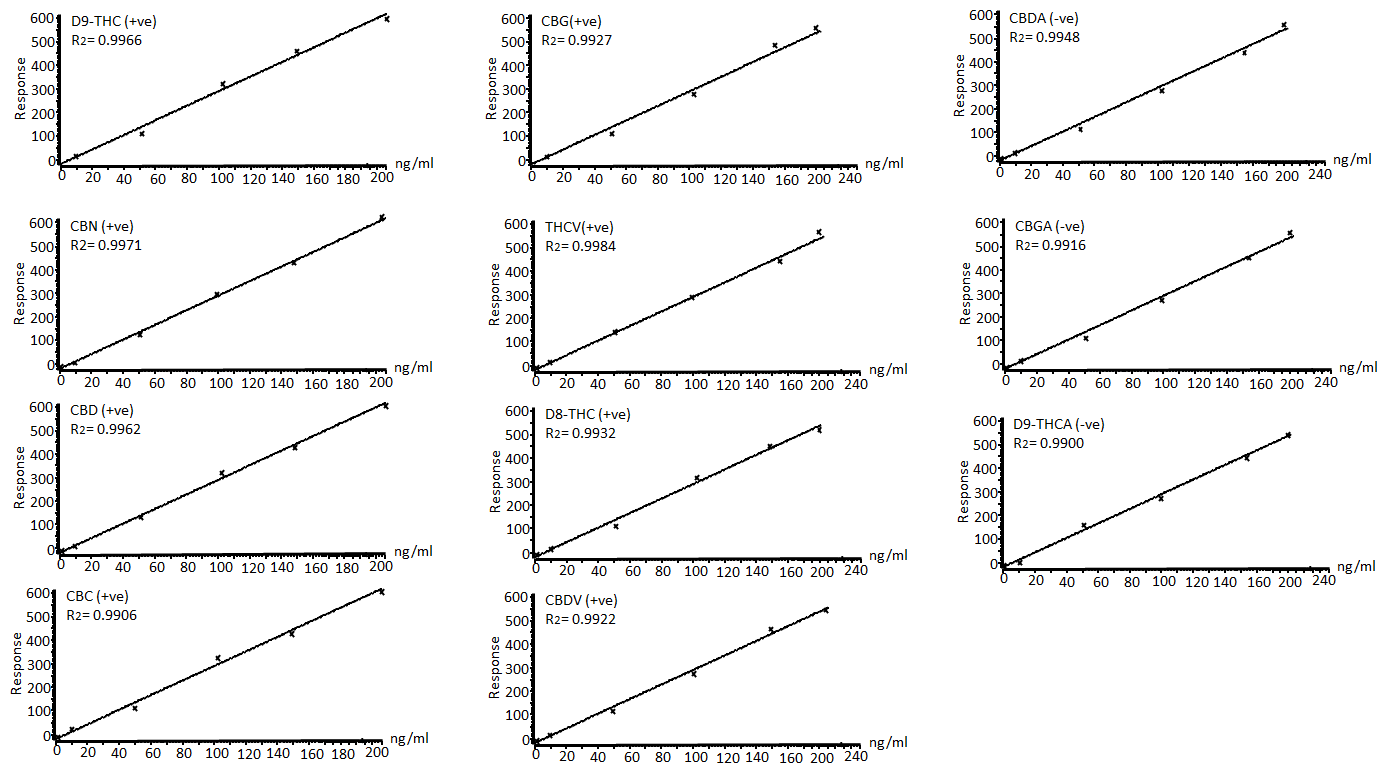


**Figure S1.** Calibration curves for 11 different target cannabinoids; a best-fit was selected for linear regression for calibration curve quantification.

**Table S1.** Matrix effect, extraction recovery of cannabinoids in samples (n=5).

| No. | Compound | Matrix effect (%)  (n=5) | | | Recovery effect (%)  (n= 5) | | |
| --- | --- | --- | --- | --- | --- | --- | --- |
|  |  | LOQ | MOQ | HOQ | LOQ | MOQ | HOQ |
| 1 | Δ^9^ THCA | 86.02131 | 102.1277 | 100.7203 | 103.8612 | 99.29563 | 98.83107 |
| 2 | CBDA | 109.0575 | 105.3236 | 94.85469 | 98.79760 | 101.3986 | 99.16057 |
| 3 | CBGA | 89.16667 | 98.58949 | 95.30389 | 99.74222 | 100.4052 | 98.80204 |
| 4 | CBG | 110.8807 | 99.33633 | 116.0664 | 99.54491 | 104.6276 | 99.97514 |
| 5 | CBD | 102.9615 | 100.4051 | 91.92202 | 95.21277 | 99.69491 | 100.1846 |
| 6 | THCV | 102.4593 | 99.77023 | 101.8441 | 97.97338 | 98.44045 | 98.88971 |
| 7 | CBN | 100.0200 | 98.36181 | 95.85626 | 95.03395 | 97.19333 | 99.80557 |
| 8 | Δ^8^ THC | 107.3693 | 104.1000 | 110.1894 | 111.4478 | 97.36602 | 91.9883 |
| 9 | Δ^9^ THC | 100.2807 | 101.81124 | 108.1627 | 110.76889 | 105.8895 | 102.3063 |
| 10 | CBC | 108.3621 | 89.68326 | 98.61793 | 97.255370 | 101.7457 | 99.55316 |
| 11 | CBDV | 107.8351 | 102.1978 | 100.4622 | 94.302100 | 98.95406 | 97.75887 |

LOQ: Lower of Quantification, MOQ: Middle of Quantification, HOQ: High of Quantification.
